# pH and Reduction Dual-Responsive Nanogels as Smart Nanocarriers to Resist Doxorubicin Aggregation

**DOI:** 10.3390/molecules27185983

**Published:** 2022-09-14

**Authors:** Ali Maruf, Małgorzata Milewska, Anna Lalik, Ilona Wandzik

**Affiliations:** 1 Department of Organic Chemistry, Bioorganic Chemistry and Biotechnology, Faculty of Chemistry, Silesian University of Technology, Krzywoustego 4, 44-100 Gliwice, Poland; 2 Biotechnology Center, Silesian University of Technology, Krzywoustego 8, 44-100 Gliwice, Poland; 3 Department of Systems Biology and Engineering, Faculty of Automatic Control, Electronics and Computer Science, Silesian University of Technology, Akademicka 16, 44-100 Gliwice, Poland

**Keywords:** doxorubicin, drug delivery, glutathione, nanogel, stimuli-responsive

## Abstract

The use of smart nanocarriers that can modulate therapeutic release aided by biological cues can prevent undesirable cytotoxicity caused by the premature release of cytotoxic drugs during nanocarrier circulation. In this report, degradable nanocarriers based on pH/reduction dual-responsive nanogels were synthesized to encapsulate doxorubicin hydrochloride (DOX) and specifically boost the release of DOX in conditions characteristic of the cancer microenvironment. Nanogels containing anionic monomer 2-carboxyethyl acrylate (CEA) and *N,N*′-bis(acryloyl)cystamine (CBA) as a degradable crosslinker have been successfully synthesized via photoinitiated free radical polymerization. The loading process was conducted after polymerization by taking advantage of the electrostatic interaction between the negatively charged nanogels and the positively charged DOX. In this case, a high drug loading capacity (DLC) of up to 27.89% was achieved. The entrapment of DOX into a nanogel network could prevent DOX from aggregating in biological media at DOX concentrations up to ~160 µg/mL. Anionic nanogels had an average hydrodynamic diameter (d_H_) of around 90 nm with a negative zeta (ζ) potential of around −25 mV, making them suitable for targeting cancer tissue via the enhanced permeation effect. DOX-loaded nanogels formed a stable dispersion in different biological media, including serum-enriched cell media. In the presence of glutathione (GSH) and reduced pH, drug release was enhanced, which proves dual responsivity. An in vitro study using the HCT 116 colon cancer cell line demonstrated the enhanced cytotoxic effect of the NG-CBA/DOX-1 nanogel compared to free DOX. Taken together, pH/reduction dual-responsive nanogels show promise as drug delivery systems for anticancer therapy.

## 1. Introduction

Doxorubicin hydrochloride (DOX), an anthracycline family antibiotic, is one of the most effective chemotherapeutic drugs developed against a variety of cancers. Free DOX, however, tends to aggregate into fibril-like structures under physiological conditions, has poor bioavailability, and causes cardiotoxicity; therefore several nanocarriers containing DOX have been developed [1]. DOX-loaded PEGylated liposomes (Doxil) became the first therapeutic nanomedicine on the market with the FDA approval in 1995. More recently, two liposomal formulations have been approved (Lipodox and Myocet) and others are under clinical trials. Liposomal DOX formulations improved circulation time and reduced cardiotoxicity but low cellular uptake in the tumor microenvironment and a poor DOX release profile from the particles are still issues [2]. In the search for new carriers, polymer-based DOX nano delivery systems have drawn attention, and some of them have reached clinical trials. Among polymeric carriers, nano-scaled hydrogel platforms play a crucial role because they are biocompatible, have a high loading capacity, and possess a potential responsivity to environmental stimuli [3,4,5,6,7,8]. These characteristics make polymer-based nanodelivery systems widely studied as tumor drug carriers, with DOX being the most intensively studied [1]. Polysaccharide-based [9] and composite [10] nanogels have also been investigated for drug delivery applications. However, nanogels based on synthetic polymers offer better opportunities for surface modification and conjugation with ligands to target tumors. Several approaches have been utilized to fabricate bioresponsive nanogels of synthetic origin that are capable of facile loading and triggering release of DOX in response to the various biological signals specific to tumor microenvironments or the intracellular compartments of cancer cells. By combining several stimuli-responsive monomers and a suitable degradable crosslinker, dual-responsive or even multiresponsive DOX delivery systems can be produced, such as pH/temperature [11,12,13,14,15], pH/redox [16,17,18,19,20,21,22,23], or pH/temperature/redox [24].

Among the various stimuli, pH and reductive environments caused by the presence of glutathione (GSH) are the most prominent and characteristically distinctive stimuli in cancer cells. The pH of the tumor tissue (pH 6.4–7.0) is lower than that of healthy tissues (pH 7.2–7.5) [25]. Moreover, pH variations in the extracellular compartments of cells (pH 7.4) compared to the intracellular lysosomes (pH 4.5−5.0) [26] can provide a pH-responsive drug release. High concentrations of GSH reaching millimolar levels (2–10 mM) within cells and micromolar levels (2–20 μM) in the blood plasma [27] facilitate the cleavage of disulfide bonds in intracellular compartments. Therefore, nanogels crosslinked by disulfide bonds are among the most extensively studied.

At acidic conditions, the controlled self-assembly of carboxyl-containing anionic nanogels with oppositely charged DOX can produce nanocarriers with a high drug loading and pH-sensitive drug release due to the decreased electrostatic interactions with the drug [16,21,22,24]. Another strategy for ensuring pH-sensitivity is the incorporation of protonable amino groups into the nanogel network since they are prone to swell at slightly acidic pH and thus can trigger drug release. In such a case, DOX is usually encapsulated by hydrophobic interactions, which results in a substantially lower DLC. The examples are nanogels containing tertiary amines [17,23].

In the previously mentioned examples of pH/reduction dual-responsive nanogels, DOX was incorporated by physical entrapment, mostly by controlled self-assembly or due to hydrophobic interactions. The drug was loaded during polymerization [19,20] or in the post-polymerization process [16,17,21,22,23,24]. In contrast, the Haag group developed pH/reduction dual-responsive nanogels where DOX was covalently conjugated to the biodegradable nanogel matrix via an acid-labile hydrazone linker and achieved a DLC of ~4 wt% [18].

The most effective way to obtain high DLCs relies on the controlled self-assembly of anionic polyelectrolyte-based nanogels with the positively charged DOX amine group. Free DOX itself is prone to aggregate under physiological conditions, which can influence the colloidal stability of nanocomplexes. By increasing the DOX content (DLC), it is more likely that nanogels will not be stable at high concentrations, which are demanded during the preparation of stock solutions for both in vitro and in vivo study purposes. The colloidal stability of DOX-loaded nanogels is usually not fully explained or not addressed. In light of the foregoing, this study was conducted with the main purpose of synthesizing nanogels that can encapsulate high content of DOX via an electrostatic interaction, maintain DOX-loaded nanogel stability in biological media, and enhance the DOX release profile in a cancer-like environment where the GSH level is high, and the environment is acidic. In this study, we compared the aggregation tendencies of free DOX (hydrochloride salt) with DOX complexed with nanogel carriers in physiological conditions to determine the encapsulation limit with regard to the colloidal stability of nanocarriers.

**Figure 1:**
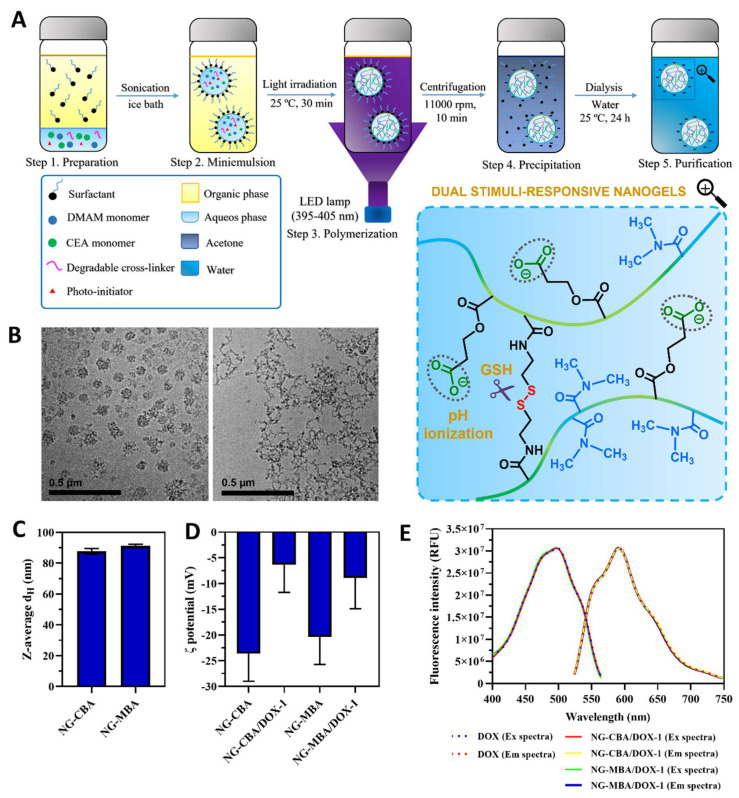
Nanogel synthesis and characterization. (**A**) A schematic diagram of the synthetic procedure of pH/reduction dual-responsive nanogels. (**B**) Cryo-TEM micrographs of NG-CBA with (right) and without (left) GSH treatment. Scale bar = 0.5 µm. (**C**) Z-average d_H_ of NG-CBA and NG-MBA nanogels in 1 mM KCl measured by DLS. Due to the interference of DOX fluorescence, DLS measurements of NG-CBA/DOX-1 and NG-MBA/DOX-1 nanogels were not possible. (**D**) ζ potential of NG-CBA, NG-CBA/DOX-1, NG-MBA, and NG-MBA/DOX-1 nanogels in 1 mM KCl. (**E**) Fluorescence spectra of DOX and NG/DOX nanogels. The data were reported as mean ± SD (*n* = 4).

In this work, nanogels were synthesized via photoinitiated free radical polymerization (FRP) of *N,N*-dimethylacrylamide (DMAM) and 2-carboxyethyl acrylate (CEA) in the presence of a disulfide-type CBA crosslinker (Figure 1A). The anionic monomer, CEA, was chosen to provide the electrostatic interactions with the positively charged DOX. CBA was selected as the crosslinker to achieve accelerated drug release triggered by the high concentration of GSH in the tumor cytoplasm. Polymerization was carried out by an inverse microemulsion technique to create the nanogels, which are able to form stable dispersions in various biological media, including serum-enriched media.

## 2. Results and Discussion

### 2.1. Synthesis and Characterization of DOX-Loaded pH/Reduction Dual-Responsive Nanogels

Dual stimuli-responsive nanogels were synthesized via FRP in inverse water-in-oil (w/o) miniemulsions using a photoinitiator (LAP) under optimized light irradiation at a wavelength of 395–405 nm (Figure 1A). The monomer composition and initiator concentration were based on our previously reported synthesis of trehalose-containing nanogels to ensure the relevant size and good stability of the nanogels [28]. DMAM was chosen as the main monomer, while CBA was used as the degradable disulfide crosslinker. To ensure the negative charge of nanogels, which is required for electrostatic interaction-mediated DOX loading, CEA monomer was incorporated. In all cases, a cyclohexane/Span 80 system was used as a continuous phase, whereas a PB solution containing monomers and LAP was used as the aqueous phase. Polymerizations were accomplished under LED irradiation at room temperature for 30 min with yields in the range of 60–65% depending on composition (Table 1). Moreover, in the same procedure, the reduction-insensitive nanogel crosslinked by MBA was prepared to compare the behavior of the nanogels in a reducing or non-reducing environment. For degradable nanogels, the notation NG-CBA was used, whereas the notation NG-MBA was adopted for non-degradable nanogels (Table 1). Both NG-CBA and NG-MBA nanogels had similar average sizes of around 90 nm, determined by DLS (Table 1, Figure 1C, Figures S1 and S2). The cryo-TEM micrographs revealed a spherical shape with particle sizes similar to those of the DLS analysis (Figure 1B). Degradation induced by GSH led to the disintegration of the nanogel network, which was also observed using cryo-TEM.

DOX was loaded into nanogel in the post-polymerization process. Three different compositions were prepared, differing in the molar ratios of anionic units of nanogel to DOX (NG-CBA/DOX-1,2) and the content of anionic units (NG-CBA/DOX-3). The unloaded NG-CBA and NG-MBA nanogels showed similar ζ potentials of −23.6 and −20.4 mV, respectively (Table 1, Figure 1D and Figures S3–S8). After DOX loading, the ζ potential of the nanogels increased above −15 mV (Table 1, Figure 1D), depending on the DLC of DOX, due to the neutralizing effects of the positively charged DOX. DOX was loaded at DLCs ranging from 9.09 to 27.89% *w/w*, according to the molar ratio of anionic units of nanogel to DOX. Similar DLCs were observed in NG-CBA-DOX-1 and NG-MBA-DOX-1 nanogels due to the same molar amount of anionic CEA units in the structure.

In comparison to the similar design of nanogels with pH/reduction responsivity, the currently studied nanogels exhibited relatively satisfying DLCs (up to ~28% *w/w*). In post-polymerization loading, other pH/reduction dual-responsive nanogels were characterized by DLCs from 2.5 to 15.1% *w/w* [17,22,23,24]. They also displayed a comparable release profile, ranging from 50 to 70% within 12 h in a cancer-mimicking environment (acidic environment and high GSH levels), but only a limited release (less than 30%) under normal physiological conditions [17,22,23,24]. In contrast, covalent conjugation of DOX to pH/reduction dual-responsive nanogels resulted in a significantly lower DLC (below 5% *w/w*), but it also enabled us to limit DOX release under physiological conditions (less than 10%), while accelerating it by almost six times in a cancer-like environment [18]. Additionally, as shown in Figure 1E, the nanogels (NG-CBA and NG-MBA) did not interfere with the fluorescence property of DOX, as indicated by identical (Ex/Em) fluorescence spectra.

### 2.2. Aggregation Behavior of DOX and DOX-Loaded pH/Reduction Dual-Responsive Nanogels

It is well known that DOX (hydrochloride salt) forms fibril-shaped aggregates under physiological conditions, and this can result in a decrease in the drug’s intercellular transportation efficiency [29]. DOX solutions in PBS (pH 7.4) at concentrations above 40 µg/mL start to aggregate. Considering DOX’s IC_50_ is in the range of 0.06 to 0.50 µg/mL depending on cancer cell line [30], one would not expect aggregation to be a problem. However, stock solutions are usually two orders of magnitude greater for biological assays. As a result, aggregation may cause a disturbance in reproducibility when a drug solution is dispensed. Therefore, aggregation of DOX in solutions should always be avoided. A substantial amount of DOX encapsulated in nanogel can also induce the aggregation of supramolecular structures formed after complexation. Our study revealed that DOX entrapment into the nanogel network could prevent aggregation at concentrations up to 160 µg/mL.

**Figure 2:**
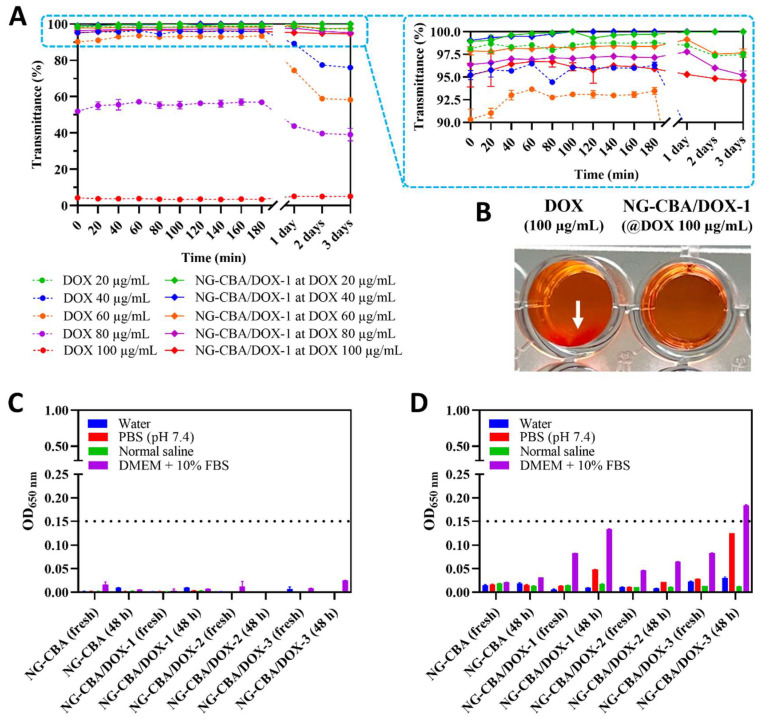
(**A**) DOX aggregation tendency in 10 mM PB (pH 7.4) at different concentrations (20, 40, 60, 80, and 100 µg/mL) compared to NG-CBA/DOX-1 nanogels (20, 40, 60, 80, and 100 µg/mL DOX) for 3 days at 37 °C. (**B**) Visual appearance of DOX 100 µg/mL and NG-CBA/DOX-1 nanogel containing 100 µg/mL DOX in 10 mM PB (pH 7.4) after 1 day at 37 °C. The white arrow indicates DOX aggregates. (**C**,**D**) Stability of NG-CBA, NG-CBA/DOX-1, NG-CBA/DOX-2, and NG-CBA-DOX-3 nanogels at concentrations of (**C**) 100 µg/mL and (**D**) 1000 µg/mL in different biological media for 48 h at 37 °C by OD 650 nm. Data were presented as mean ± SD (*n* = 4).

Figure 2A,B shows how the NG-CBA/DOX-1 nanogel improved the solubility of DOX in 10 mM PB (pH 7.4). The transmittance value of free DOX at a concentration of 20 µg/mL was nearly 100% over 3 days which indicates that the solution is free of insoluble aggregates. However, the transmittance value decreased over time at concentrations of 40–80 µg/mL, indicating DOX aggregation (Figure 2A). DOX instantly aggregated and sedimented at a concentration of 100 µg/mL (Figure 2B). Moreover, at this DOX concentration, the transmittance value was nearly zero. In contrast, the NG-CBA/DOX-1 nanogel containing 100 µg/mL of DOX exhibited a transmittance value of 95%, which proves the lack of aggregation. The stability of NG-CBA and NG-CBA/DOX-1,2,3 nanogels was further studied in different biological media, including serum-enriched media, where nanoparticle dispersion might collapse and form aggregates. Figure 2C showed that both bare nanogel and DOX-containing nanogels were stable for at least 2 days in different biological media at a nanogel concentration of 100 µg/mL. In the higher nanogel concentration of 1000 µg/mL, the unloaded NG-CBA nanogel was stable in all tested media. However, the DOX-containing nanogels showed limited stability in serum-enriched media for both fresh solutions and solutions incubated at 37 °C; for 2 days (Figure 2D). The greater the DOX content, the greater the decrease in transparency. Therefore, in order to avoid any aggregation, NG-CBA-DOX-1 nanogel containing only 16.29 wt% of DOX was used for further drug release studies.

### 2.3. DOX Release Study

**Figure 3:**
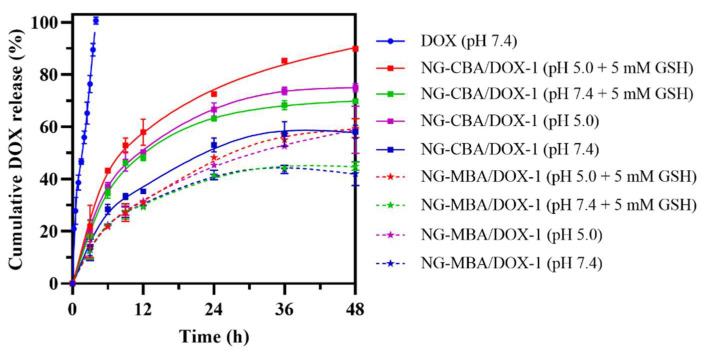
Drug release profile from NG-CBA/DOX-1 nanogels (squares and solid lines) and NG-MBA/DOX-1 nanogels (stars and dotted lines) in four solutions: GSH (0 and 5 mM) in PBS (pH 7.4 and 5.0). The nanogel concentrations were 250 µg/mL and the solution temperature was 37 °C. The data were presented as mean ± SD (*n* = 4).

The drug release behaviors of nanogels containing DOX were investigated with and without GSH at pH values of 7.4 and 5.0 using the dialysis method. The cumulative release percentages of DOX-loaded in the nanogels versus time are plotted in Figure 3. In the absence of GSH, less than 33% of loaded DOX was released from the NG-CBA/DOX-1 nanogel in 12 h at pH 7.4. The DOX release was enhanced by GSH and in the presence of 5 mM GSH, nearly 50% of the loaded DOX was released within 12 h. Additional enhancement in DOX release was observed at pH 5.0 where the cumulative release percentage after 12 h reached 50 and 58% in the absence and presence of 5 mM GSH, respectively. Larger differences became apparent after 48 h, when almost complete release of DOX from NG-CBA-DOX-1 was observed at pH 5.0 in 5 mM GSH, while only 50% of DOX release occurred at pH 7.4 without reducing the environment.

As expected, in the case of the NG-MBA/DOX-1 nanogel, which contains the reduction-insensitive crosslinker, no significant changes in DOX release were observed in the presence and absence of GSH. In turn, decreasing the pH of the environment from 7.4 to 5.0 increased the DOX release rate and the cumulative DOX release reached about 40 and 55%, respectively.

### 2.4. Cytotoxicity Study in Cancer Cell Line

**Figure 4:**
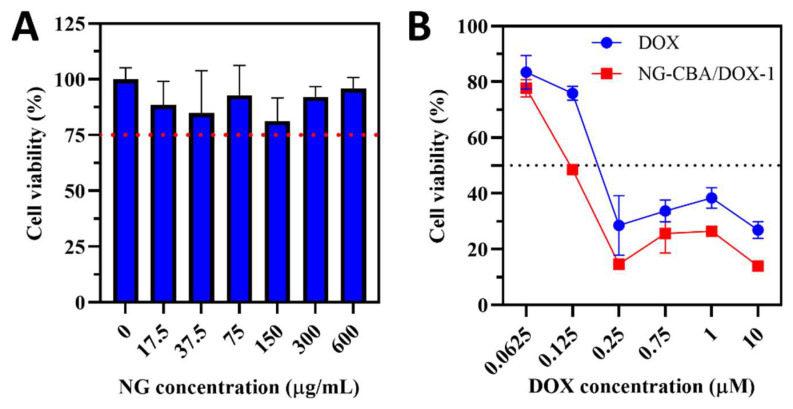
(**A**) Cytotoxicity profile of bare NG-CBA nanogels in the HCT 116 colon cancer cell line at different concentrations (17.5, 37.5, 75, 150, 300, and 600 µg/mL) after 48 h of incubation at 37 °C. (**B**) Cytotoxicity profile of DOX and NG-CBA/DOX-1 nanogel in the HCT 116 colon cancer cell line at different DOX concentrations (0.0625, 0.125, 0.25, 0.75, 1.0, and 10.0 µM) after 48 h of incubation at 37 °C. The data were presented as mean ± SD (*n* = 4).

The cytotoxicity profile of DOX and the two nanogels, NG-CBA and NG-CBA/DOX-1, was assessed in the HCT 116 colon cancer cell line. Firstly, in vitro studies revealed that the unloaded nanogel NG-CBA was not cytotoxic to HCT 116 cells after incubation at different concentrations ranging from 17.5 to 600 µg/mL (Figure 4A). Notably, DOX-loaded nanogels had a higher efficiency than free DOX in causing cytotoxic effects on cancer cells (Figure 4B). Moreover, one can speculate that the rate of drug release from GSH-mediated degradable nanogels will be even greater in cells with much higher GSH levels, such as breast or ovarian cancer cells [31].

## 3. Materials and Methods

### 3.1. Materials and Reagents

#### 3.1.1. General Methods

Miniemulsion formation and nanogel redispersion were accomplished by ultrasonication using Sonics VCX 130 (Sonics & Materials, Inc., Newtown, CT, USA) using 60% and 40% amplitudes, respectively. Lyophilization of purified nanogels and DOX-loaded nanogels from frozen samples (in water, at −80 °C) was carried out under 0.035 mbar at −50 °C (ALPHA 1-2 LDplus, CHRIST). A SpectraMax i3x Multi-Mode Microplate Reader (Molecular Devices, USA) was used for stability, cytotoxicity, and fluorescence assays. Phosphate buffered saline (PBS), phosphate buffer (PB), and normal saline solutions were freshly prepared. Deionized water (DI water) was produced using a reverse osmosis system (conductivity < 2 μS/cm).

#### 3.1.2. Materials and Reagents for pH/Reduction Dual-Responsive Nanogels Synthesis

Doxorubicin hydrochloride (DOX, Cayman Chemical), (*N,N*-dimethylacrylamide (DMAM, Sigma Aldrich, Burlington, MA, USA), 2-carboxyethyl acrylate (CEA, Sigma Aldrich), *N,N*′-bis(acryloyl)cystamine (CBA, Alfa Aesar), *N,N*′-methylenebisacrylamide (MBA, Acros Organics, Geel, Belgium), lithium phenyl (2,4,6-trimethylbenzoyl)phosphinate (LAP, Carbosynth), Span 80 (Sigma Aldrich), cyclohexane (Chempur), acetone (Chempur), dimethyl sulfoxide (DMSO, Fisher Bioreagents, Pittsburgh, PA, USA), and dialysis membrane (Spectrum™ Spectra/Por™ 2 RC Dialysis Membrane, MWCO: 12–14 kDa).

#### 3.1.3. Materials and Reagents for Cell Culture and In Vitro Assays

HCT 116 colon cancer cell line (catalog no. CCL-247) was obtained from the American Type Culture Collection (ATCC, Manassas, VA, USA), Dulbecco’s Modified Eagle Medium (DMEM, PAN Biotech), Fetal Bovine Serum (FBS, PAN Biotech, Aidenbach, Germany), PBS (PAN Biotech), and CCK-8 kit (Bimake).

### 3.2. Synthesis of pH/Reduction Dual-Responsive Nanogels

pH/reduction dual-responsive nanogels were synthesized via free radical polymerization (FRP) in an inverse water-in-oil (w/o) miniemulsion. A 10:1 (*v*:*v*) water-in-oil (w/o) miniemulsion was composed of cyclohexane (10.0 mL) containing Span 80 (600 mg) as the organic continuous phase; the aqueous phase (1.0 mL) consisted of PB solution (pH 7.0) containing monomers and the photoinitiator LAP. The general procedure for the synthesis of nanogels was as follows (Figure 1A). Briefly, the aqueous phase was prepared in a 4 mL dark vial by adding CBA (26.0 mg, 0.10 mmol) and 0.2 M PB (pH 7.0) containing DMSO (10% *v/v*) with the main monomers: DMAM and CEA (amounts specified in Table 1). The mixture was vortexed for approximately 10–15 min to completely dissolve all the monomers. Finally, the solution of LAP initiator (2.3 mg, 0.008 mmol) was added. Then, the aqueous phase was transferred into a 20 mL glass vial containing a cold organic phase (4 °C) and sonicated at 60% amplitude for 5 min to create a miniemulsion. The vial was then wrapped with aluminum foil and photoirradiated from the bottom of the vial with High Power Light-Emitting Diodes (LEDs, 3 W, 395–405 nm) for 30 min. Nanogels were precipitated in cold acetone (40 mL), centrifuged at 11,000 rpm for 10 min, and washed twice with acetone. After air drying overnight, crude nanogels were redispersed in DI water and dialyzed against water for 24 h using a dialysis membrane (MWCO 12–14 kDa) with multiple media changes. Pure nanogel dispersions (after dialysis) were frozen at −80 °C and lyophilized to obtain the nanogel powder. The MBA-crosslinked nanogels were synthesized using the same procedure as mentioned above, except CBA was replaced with MBA at the same molar ratio (Table 1).

### 3.3. Loading of DOX into Anionic Nanogels

Post-polymerization loading of DOX into the nanogels was accomplished as follows. Initially, 5.0 mg of dry nanogel was placed in a 4 mL glass vial. Then, the DOX solution (1.0 mg of DOX in 800 µL of water) was added. After the nanogels swelled (approximately 5 min), the suspension was stirred in the dark overnight at 400 rpm. Afterward, the excess DOX was eliminated by dialysis using a dialysis membrane (MWCO 12–14 kDa) against DI water with multiple media changes. Finally, pure DOX-loaded nanogels were lyophilized and stored as nanogel powder.

### 3.4. Nanogel Characterization

#### 3.4.1. Measurement of Drug Loading Efficiency (DLE) and Drug Loading Capacity (DLC)

The measurements of drug loading efficiency (DLE) and drug loading capacity (DLC) of nanogels were based on the fluorescence intensity using the standard curve of *DOX* in water (Ex/Em max of 498/593 nm, R^2^ = 0.997). According to the fluorescence spectra, both *DOX* and *DOX-loaded* nanogels share identical fluorescence spectra with the same Ex/Em max (Figure 1E). Following measurement of its fluorescence intensity, *DOX-loaded* nanogels were dispersed in DI water and diluted to a final concentration of 10.0 µg/mL. DLE and DLC were then calculated using the following equations:

$$\mathrm{DLE}\;(\%)=\frac{actual\;loaded\;DOX}{Initial\;feed\;of\;DOX}\;\times \;100$$

$$\mathrm{DLC}\;(\%)=\frac{actual\;loaded\;DOX}{DOX-loaded\;nanogel}\;\times \;100$$

The Z-average hydrodynamic diameter (d_H_) and polydispersity index (PdI) of DOX-loaded nanogels (1.0 mg/mL) in 1 mM KCl solution or PBS (pH 7.4) were determined using a dynamic light scattering (DLS, Malvern, Zetasizer Nano 90S) system equipped with a 4 mV He–Ne ion laser (λ = 633 nm) as the light source at a scattering angle of 90°. All samples were diluted from stock (10 mg/mL, prepared with sonication at 40% amplitude for 30 s) to the desired media concentration (1.0 mg/mL) without additional sonication. The ζ potentials of bare nanogels and DOX-loaded nanogels were measured using electrophoretic light scattering (ELS) measurements (Malvern, Zetasizer Nano ZC) in 1 mM KCl solution.

#### 3.4.2. Cryogenic Transmission Electron Microscopy (cryo-TEM)

GSH-treated and untreated nanogels (500 µg/mL) in DI water were observed under cryo-TEM using a Tecnai F20 X TWIN microscope (FEI Company, Hillsboro, Oregon, USA). Specimens were prepared via the vitrification of aqueous solutions on oxygen plasma-activated grids with holey carbon film (Quantifoil R 2/2; Quantifoil Micro Tools GmbH, Großlöbichau, Germany).

#### 3.4.3. Measurement of DOX and DOX-Loaded Nanogel Aggregation in Biological Media

The aggregation of DOX was observed in 10 mM PB (pH 7.4) within 3 days. DOX was dispersed in 10 mM PB (pH 7.4) at various concentrations (20, 40, 60, 80, and 100 g/mL) and placed in a 48-well plate (volume: 1.0 mL for each concentration) and incubated at 37 °C. NG-CBA/DOX-1 at DOX concentrations of 20, 40, 60, 80, and 100 µg/mL were also dispersed in 10 mM PB (pH 7.4, stock was prepared in water with sonication at 40% amplitude for 30 s). At predetermined time intervals (0, 20, 40, 60, 80, 100, 120, 140, 160, 180, 1440, 2880, and 4320 min), the samples were observed visually and their optical densities (OD) at 650 nm were measured. The absorbance value was converted to % transmittance using the following equation:

Transmittance (%) = antilog (2 − absorbance)

#### 3.4.4. Stability Study of Nanogel in Biological Media

For the stability study, stock solutions of nanogels were prepared in water with sonication at 40% amplitude for 30 s. The colloidal stability of bare NG-CBA and NG-CBA/DOX-1,2,3 nanogels was measured at concentrations of 100 and 1000 µg/mL, low and high concentrations, respectively, in different biological media, including water, PBS (pH 7.4), normal saline (0.90% *w/v* of NaCl), and DMEM + 10% FBS at 37 °C for 48 h. NG-CBA/DOX-1,2,3 (concentration: 100 µg/mL) contained DOX at concentrations of 16.29, 9.09, and 27.89 µg/mL, respectively. Meanwhile, NG-CBA/DOX-1,2,3 (concentration: 1000 µg/mL) contained exactly 10× more DOX: 162.9, 90.9, and 278.9 µg/mL, respectively. The colloidal stability was observed by measuring OD at 650 nm in 96-well plates using a microplate reader. OD values ranging from 0.00–0.15 were considered a stable dispersion.

### 3.5. Drug Release Study

DOX release from the nanogel was analyzed using the dialysis method. Initially, a dispersion of nanogel at a concentration of 250 µg/mL in relevant PBS (pH 7.4 or 5.0) containing 5 mM GSH or without GSH additive was prepared. Then 800 µL of NG-CBA/DOX-1 or NG-MBA/DOX-1 at a DOX concentration of 40.7 μg/mL or free DOX at 40.7 μg/mL was placed into the dialysis capsule (QuixSep^®^, 1 mL) using a dialysis membrane (MWCO 12–14 kDa). The dialysis capsules were immersed in 40 mL of PBS (pH 7.4 with 0 or 5 mM GSH and pH 5.0 with 0 or 5 mM GSH) and incubated at 37 °C under continuous shaking (110 rpm). At each predetermined time interval of 3, 6, 12, 24, 36, and 48 h, 400 µL of dissolution medium was withdrawn from each vial and replaced with the same amount of pre-warmed fresh medium. The amount of released DOX in the withdrawn samples was determined by measuring its fluorescence intensity using a λ_ex_ of 498 nm and λ_em_ of 593 nm and converting it to DOX concentration using a standard curve.

### 3.6. Cytotoxicity Study

For the cytotoxicity study, stock solutions of nanogels were prepared in water with sonication at 40% amplitude for 30 s. The cytotoxicity profile of NG-CBA/DOX-1 was assessed in the HCT 116 colon cancer cell line using a standard CCK-8 assay. For 24 h, HCT 116 colon cancer cells (5 × 10^3^ cells/well) were seeded in 96-well plates in 100 μL of DMEM + 10% FBS. Then, the cells were incubated with DOX and NG-CBA/DOX-1 at various concentrations of DOX (0.0625, 0.125, 0.25, 0.75, 1.0, and 10.0 μM) for 48 h. In addition, the cytotoxicity of bare NG-CBA was also evaluated at different concentrations of nanogels (17.5, 37.5, 75, 150, 300, and 600 μg/mL). The CCK-8 assay was carried out by adding 10 μL of CCK-8 solution to each well after 2 h of incubation at 37 °C. The absorbance was then measured by a microplate reader at 450 nm. The relative cell viability (%) was expressed as a fraction of the percentage of cell growth occurring in the presence of nanogel vs. the absence of nanogel (control).

### 3.7. Statistical Analysis

GraphPad Prism Version 6.0 software (GraphPad, San Diego, CA, USA) was used for the statistical analysis. Data analysis was performed using one-way analysis of variance (ANOVA). The minimum level of significance was set at *p* < 0.05, with all data displayed as mean ± SD (*n* = 4).

## 4. Conclusions

Dual stimuli-responsive nanogels based on acrylic polymers have been produced in a facile and straightforward method. DOX-encapsulation was achieved by a facile diffusion process, forming a complex with ionized carboxylic groups of CEA units. Nanogels showed high DLCs (up to 27.89 wt%) and suitable nanoparticle size for enhanced permeation effects. More importantly, nanogels could prevent DOX from aggregating in biological media up to DOX concentrations of ~160 µg/mL (NG-CBA/DOX-1), whereas free DOX was only stable at concentrations lower than 40 µg/mL.

DOX-encapsulated degradable nanogels showed enhanced release rates at lower pH (i.e., pH of 5.0) and in a reducing environment showing an obvious pH/reduction dual-responsive controlled drug release capability. In vitro studies revealed that the bare nanogel was not cytotoxic to HCT 116 colon cancer cells, whereas DOX-containing nanogels were toxic against HCT 116 colon cancer cells with a better efficacy than free DOX. The presented nanogels were conveniently synthesized and exhibited excellent colloidal stability and cationic drug entrapment capability making them a promising alternative to existing DOX carrier systems. Additionally, nanogels can be further modified and conjugated with targeting ligands for active targeted delivery.

## Figures and Tables

**Table 1:** Monomer feed composition and physicochemical properties of nanogels.

Nanogel	Monomer Feed Composition ^a^		Post-Polymerization Loading			DLC (%)	DLE (%)	d_H_ (PdI) (nm)	ζ Potential (mV)
	CEA mg (mmol)	DMAM mg (mmol)	NG (mg)	DOX (mg)	CEA:DOX (mol:mol)				
NG-CBA	16.0 (0.111)	183.6 (1.852)	-	-	-	-	-	87.67 (0.30)	−23.6
NG-MBA	16.0 (0.111)	183.6 (1.852)	-	-	-	-	-	91.19 (0.38)	−20.4
NG-CBA/DOX-1	16.0 (0.111)	183.6 (1.852)	5.0	1.0	1:0.85	16.29	97.74	^b^	−6.33
NG-CBA/DOX-2	16.0 (0.111)	183.6 (1.852)	5.0	0.5	1:0.42	9.09	99.62	^b^	−18.6
NG-CBA/DOX-3	32.0 (0.222)	167.6 (1.691)	5.0	2.0	1:0.85	27.89	97.61	^b^	−11.3
NG-MBA/DOX-1	16.0 (0.111)	183.6 (1.852)	5.0	1.0	1:0.85	16.47	98.78	^b^	−8.92

^a^ CBA or MBA were used in an equimolar quantity of 0.10 mmol, LAP was used at a concentration of 0.008 mmol, and the total mass of the monomers was 227.9 mg and 217.2 mg for NG-CBA and NG-MBA nanogels, respectively. ^b^ DLS measurement of DOX-containing nanogels was not possible due to the interference of DOX fluorescence.

## Data Availability

Not applicable.

## Supplementary Materials

The following supporting information can be downloaded at: https://www.mdpi.com/article/10.3390/molecules27185983/s1, Figure S1: Average size distribution (d_H_) of NG-CBA, (*n* = 4); Figure S2: Average size distribution (d_H_) of NG-MBA, (*n* = 4); Figure S3: Average zeta potential of NG-CBA, (*n* = 4); Figure S4: Average zeta potential of NG-MBA, (*n* = 4); Figure S5: Average zeta potential of NG-CBA/DOX-1, (*n* = 4); Figure S6: Average zeta potential of NG-MBA/DOX-1, (*n* = 4); Figure S7: Average zeta potential of NG-CBA/DOX-2, (*n* = 4); Figure S8: Average zeta potential of NG-CBA/DOX-3, (*n* = 4).

## References

[1] Kanwal U., Irfan Bukhari N., Ovais M., Abass N., Hussain K., Raza A. (2018). Advances in nano-delivery systems for doxorubicin: An updated insight. J. Drug Target..

[2] Alyane M., Barratt G., Lahouel M. (2016). Remote loading of doxorubicin into liposomes by transmembrane pH gradient to reduce toxicity toward H9c2 cells. Saudi Pharm. J..

[3] Kabanov A.V., Vinogradov S.V. (2009). Nanogels as pharmaceutical carriers: Finite networks of infinite capabilities. Angew. Chem. Int. Ed. Engl..

[4] Li C., Obireddy S.R., Lai W.F. (2021). Preparation and use of nanogels as carriers of drugs. Drug Deliv..

[5] Huang D., Qian H., Qiao H., Chen W., Feijen J., Zhong Z. (2018). Bioresponsive functional nanogels as an emerging platform for cancer therapy. Expert Opin. Drug Deliv..

[6] Vashist A., Kaushik A., Vashist A., Bala J., Nikkhah-Moshaie R., Sagar V., Nair M. (2018). Nanogels as potential drug nanocarriers for CNS drug delivery. Drug Discov. Today.

[7] Neamtu I., Rusu A.G., Diaconu A., Nita L.E., Chiriac A.P. (2017). Basic concepts and recent advances in nanogels as carriers for medical applications. Drug Deliv..

[8] Zhang H., Zhai Y., Wang J., Zhai G. (2016). New progress and prospects: The application of nanogel in drug delivery. Mater. Sci. Eng. C.

[9] Debele T.A., Mekuria S.L., Tsai H.C. (2016). Polysaccharide based nanogels in the drug delivery system: Application as the carrier of pharmaceutical agents. Mater. Sci. Eng. C.

[10] Mohammadi M., Arabi L., Alibolandi M. (2020). Doxorubicin-loaded composite nanogels for cancer treatment. J. Control. Release.

[11] Chiang W.H., Ho V.T., Huang W.C., Huang Y.F., Chern C.S., Chiu H.C. (2012). Dual stimuli-responsive polymeric hollow nanogels designed as carriers for intracellular triggered drug release. Langmuir.

[12] Dhanya S., Bahadur D., Kundu G.C., Srivastava R. (2013). Maleic acid incorporated poly-(N-isopropylacrylamide) polymer nanogels for dual-responsive delivery of doxorubicin hydrochloride. Eur. Polym. J..

[13] Chiang W.H., Huang W.C., Chang Y.J., Shen M.Y., Chen H.H., Chern C.S., Chiu H.C. (2014). Doxorubicin-Loaded Nanogel Assemblies with pH/Thermo-triggered Payload Release for Intracellular Drug Delivery. Macromol. Chem. Phys..

[14] Peng H., Huang X., Oppermann A., Melle A., Weger L., Karperien M., Wöll D., Pich A. (2016). A facile approach for thermal and reduction dual-responsive prodrug nanogels for intracellular doxorubicin delivery. J. Mater. Chem. B.

[15] Rao K.M., Suneetha M., Kumar D.V., Kim H.J., Seok Y.J., Han S.S. (2022). Dual Responsive poly(vinyl caprolactam)-Based Nanogels for Tunable Intracellular Doxorubicin Delivery in Cancer Cells. Pharmaceutics.

[16] Pan Y.J., Chen Y.Y., Wang D.R., Wei C., Guo J., Lu D.R., Chu C.C., Wang C.C. (2012). Redox/pH dual stimuli-responsive biodegradable nanohydrogels with varying responses to dithiothreitol and glutathione for controlled drug release. Biomaterials.

[17] Li M., Tang Z., Sun H., Ding J., Song W., Chen X. (2013). pH and reduction dual-responsive nanogel cross-linked by quaternization reaction for enhanced cellular internalization and intracellular drug delivery. Polym. Chem..

[18] Zhang X., Achazi K., Steinhilber D., Kratz F., Dernedde J., Haag R. (2014). A facile approach for dual-responsive prodrug nanogels based on dendritic polyglycerols with minimal leaching. J. Control. Release.

[19] Sousa-Herves A., Wedepohl S., Calderón M. (2015). One-pot synthesis of doxorubicin-loaded multiresponsive nanogels based on hyperbranched polyglycerol. Chem. Commun..

[20] Chen W., Achazi K., Schade B., Haag R. (2015). Charge-conversional and reduction-sensitive poly(vinyl alcohol) nanogels for enhanced cell uptake and efficient intracellular doxorubicin release. J. Control. Release.

[21] Wu H., Jin H., Wang C., Zhang Z., Ruan H., Sun L., Yang C., Li Y., Qin W., Wang C. (2017). Synergistic Cisplatin/Doxorubicin Combination Chemotherapy for Multidrug-Resistant Cancer via Polymeric Nanogels Targeting Delivery. ACS Appl. Mater. Interfaces.

[22] Cuggino J.C., Gatti G., Picchio M.L., Maccioni M., Gugliotta L.M., Alvarez Igarzabal C.I. (2019). Dually responsive nanogels as smart carriers for improving the therapeutic index of doxorubicin for breast cancer. Eur. Polym. J..

[23] Kumar P., Behl G., Kaur S., Yadav N., Liu B., Chhikara A. (2021). Tumor microenvironment responsive nanogels as a smart triggered release platform for enhanced intracellular delivery of doxorubicin. J. Biomater. Sci. Polym. Ed..

[24] Zhan Y., Gonçalves M., Yi P., Capelo D., Zhang Y., Rodrigues J., Liu C., Tomás H., Li Y., He P. (2015). Thermo/redox/pH-triple sensitive poly(N-isopropylacrylamide-co-acrylic acid) nanogels for anticancer drug delivery. J. Mater. Chem. B.

[25] Hao G., Xu Z.P., Li L. (2018). Manipulating extracellular tumour pH: An effective target for cancer therapy. RSC Adv..

[26] Zeng J., Shirihai O.S., Grinstaff M.W. (2020). Modulating lysosomal pH: A molecular and nanoscale materials design perspective. J. Life Sci..

[27] Cheng R., Feng F., Meng F., Deng C., Feijen J., Zhong Z. (2011). Glutathione-responsive nano-vehicles as a promising platform for targeted intracellular drug and gene delivery. J. Control. Release.

[28] Maruf A., Milewska M., Kovács T., Varga M., Vellai T., Lalik A., Student S., Borges O., Wandzik I. (2022). Trehalose-releasing nanogels: A step toward a trehalose delivery vehicle for autophagy stimulation. Biomater. Adv..

[29] Zhu L., Yang S., Qu X., Zhu F., Liang Y., Liang F., Wang Q., Li J., Li Z., Yang Z. (2014). Fibril-shaped aggregates of doxorubicin with poly-L-lysine and its derivative. Polym. Chem..

[30] Silva V.R., Corrêa R.S., Santos L.S., Soares M., Batista A.A., Bezerra D.P. (2018). A ruthenium-based 5-fluorouracil complex with enhanced cytotoxicity and apoptosis induction action in HCT116 cells. Sci. Rep..

[31] Gamcsik M.P., Kasibhatla M.S., Teeter S.D., Colvin O.M. (2012). Glutathione levels in human tumors. Biomarkers.

