# Supplementary material for: pH and Reduction Dual-Responsive Nanogels as Smart Nanocarriers to Resist Doxorubicin Aggregation
Source: Molecules. 2022 Sep 14;27(18):5983. doi: 10.3390/molecules27185983 (PMC9505369; doi:10.3390/molecules27185983)
Supplement: Supplementary file 1 [file molecules-27-05983-s001.zip › molecules-1895562-supplementary.pdf]

Supplementary Information

DLS measurements of nanogels

			Size (d.nm):	% Intensity:	St Dev (d.nm):
Z-Average (d.nm):	87.67	Peak 1:	117.8	91.6	63.37
PdI:	0.307	Peak 2:	20.87	5.5	4.801
Intercept:	0.965	Peak 3:	4238	2.9	1293
Result quality :	Good				

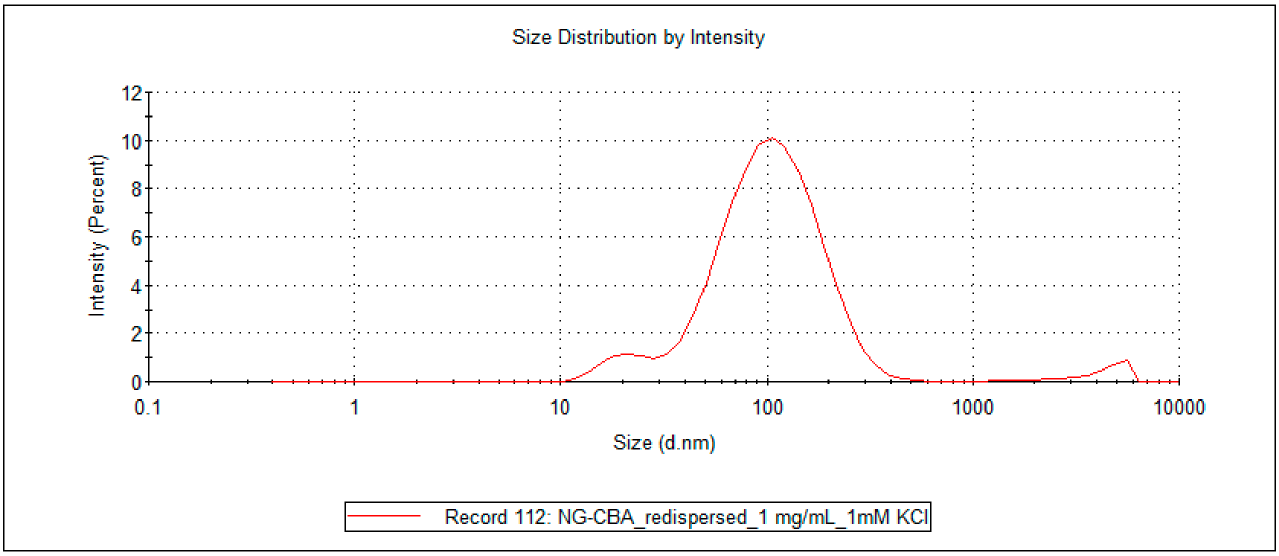

Figure S1. Average size distribution (d_H) of NG-CBA, (n = 4).

			Size (d.nm):	% Intensity:	St Dev (d.nm):
Z-Average (d.nm):	91.19	Peak 1:	142.3	83.8	73.17
PdI:	0.380	Peak 2:	33.37	14.8	7.334
Intercept:	0.968	Peak 3:	5078	1.4	563.0
Result quality :	Good				

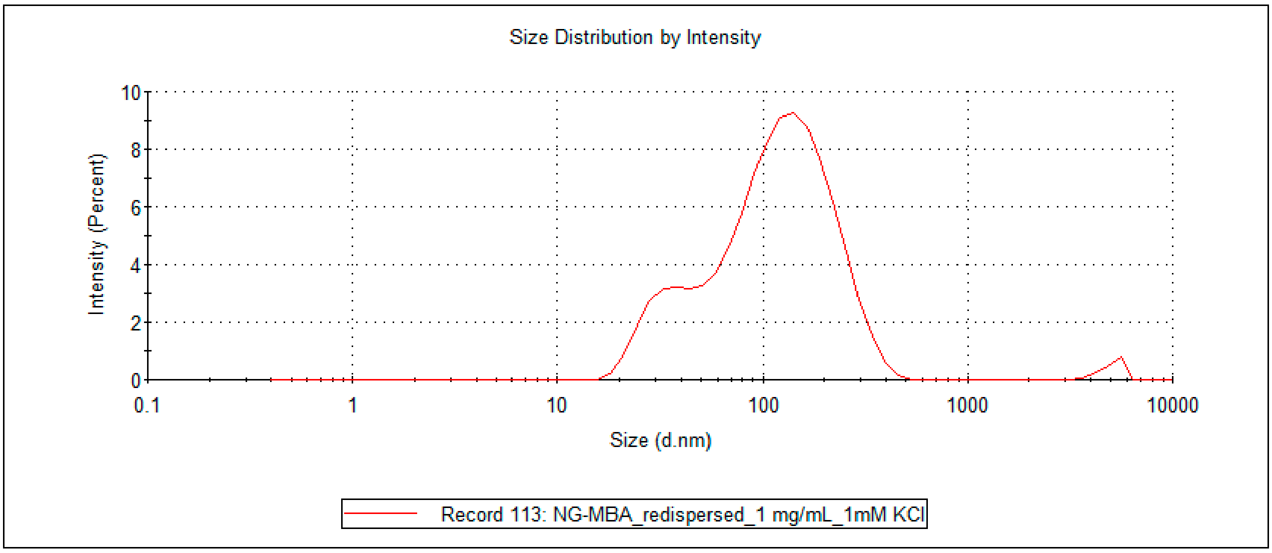

Figure S2. Average size distribution (d_H) of NG-MBA, (n = 4).

Zeta potential measurements of nanogels

			Mean (mV)	Area (%)	St Dev (mV)
Zeta Potential (mV):	-23.6	Peak 1:	-23.5	100.0	5.70
Zeta Deviation (mV):	5.40	Peak 2:	0.00	0.0	0.00
Conductivity (mS/cm):	0.203	Peak 3:	0.00	0.0	0.00
Result quality :	Good				

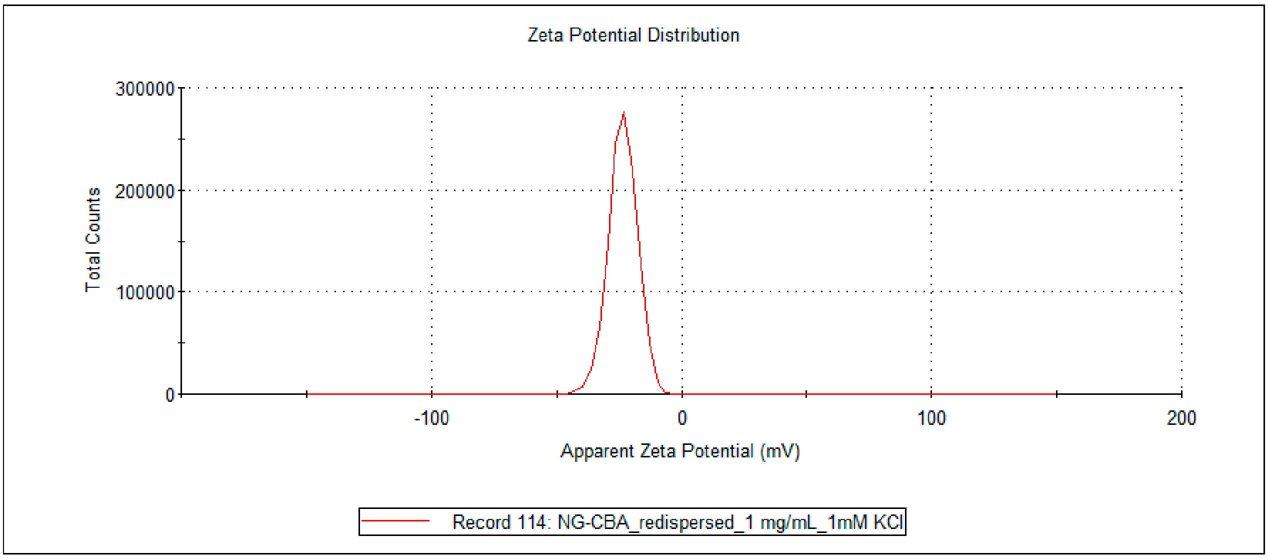

Figure S3. Average zeta potential of NG-CBA, (n = 4).

			Mean (mV)	Area (%)	St Dev (mV)
Zeta Potential (mV):	-20.4	Peak 1:	-20.3	100.0	5.40
Zeta Deviation (mV):	5.35	Peak 2:	0.00	0.0	0.00
Conductivity (mS/cm):	0.182	Peak 3:	0.00	0.0	0.00
Result quality :	Good				

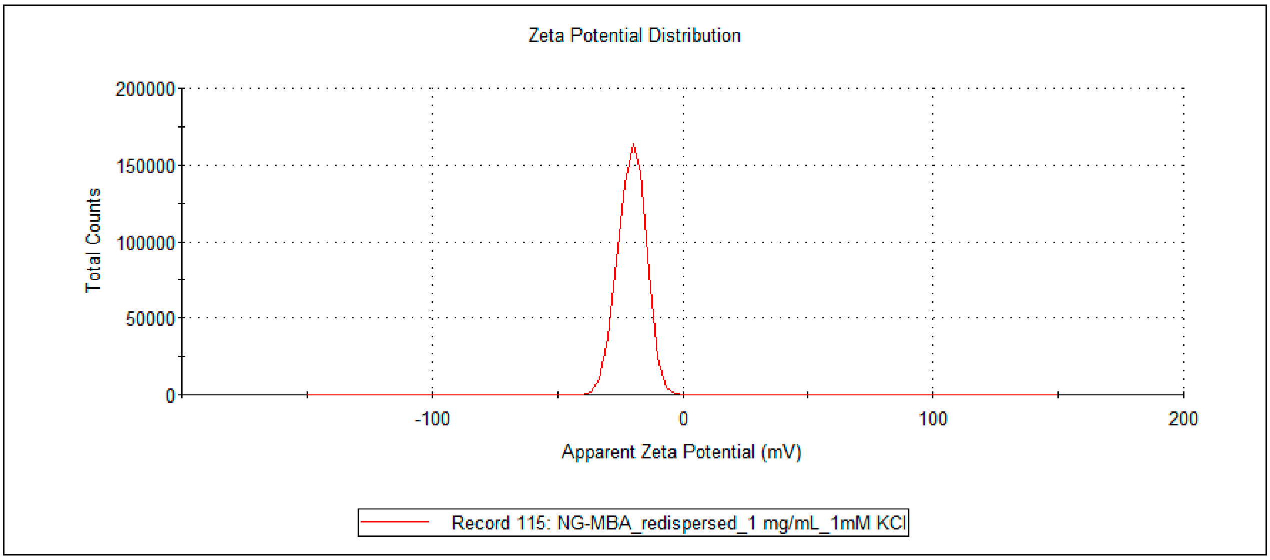

Figure S4. Average zeta potential of NG-MBA, (n = 4).

			Mean (mV)	Area (%)	St Dev (mV)
Zeta Potential (mV):	-6.33	Peak 1:	-6.19	100.0	5.82
Zeta Deviation (mV):	5.42	Peak 2:	0.00	0.0	0.00
Conductivity (mS/cm):	0.163	Peak 3:	0.00	0.0	0.00
Result quality :	Good				

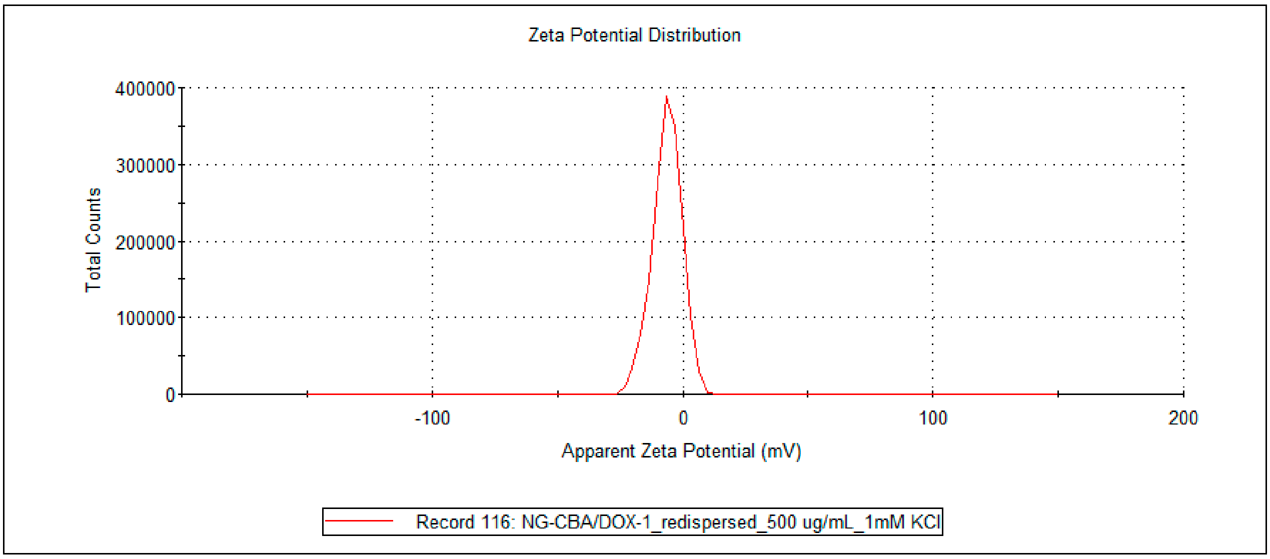

Figure S5. Average zeta potential of NG-CBA/DOX-1, (*n* = 4).

			Mean (mV)	Area (%)	St Dev (mV)
Zeta Potential (mV):	-8.92	Peak 1:	-9.03	100.0	6.26
Zeta Deviation (mV):	5.98	Peak 2:	0.00	0.0	0.00
Conductivity (mS/cm):	0.171	Peak 3:	0.00	0.0	0.00
Result quality :	Good				

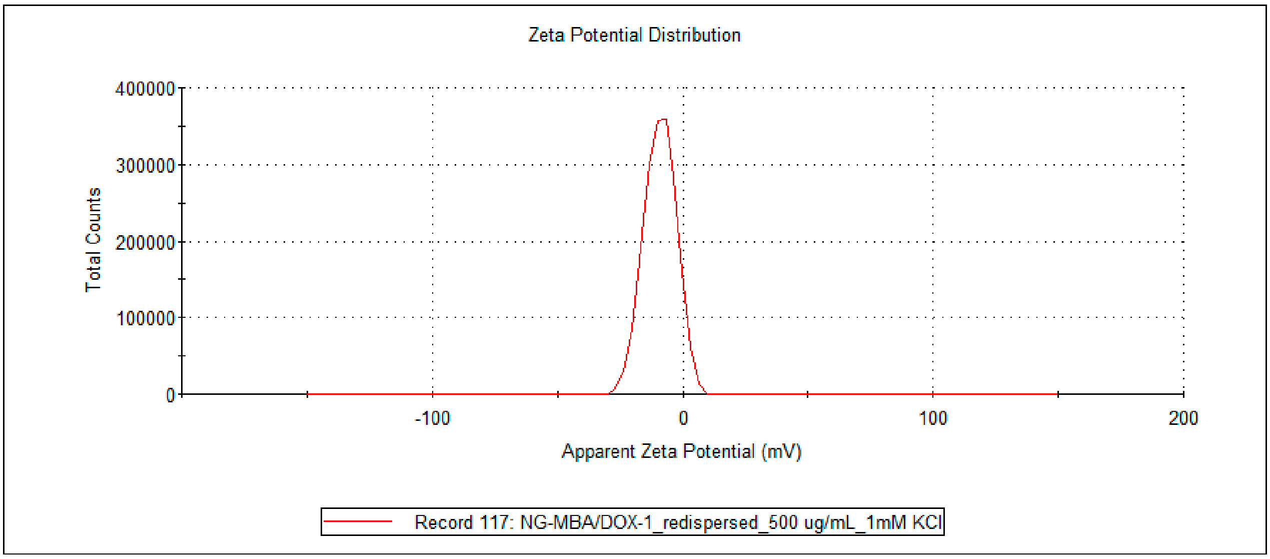

Figure S6. Average zeta potential of NG-MBA/DOX-1, (*n* = 4).

			Mean (mV)	Area (%)	St Dev (mV)
Zeta Potential (mV):	-18.6	Peak 1:	-18.5	99.9	5.06
Zeta Deviation (mV):	5.16	Peak 2:	6.46	0.1	0.00
Conductivity (mS/cm):	0.218	Peak 3:	0.00	0.0	0.00

Result quality : Good

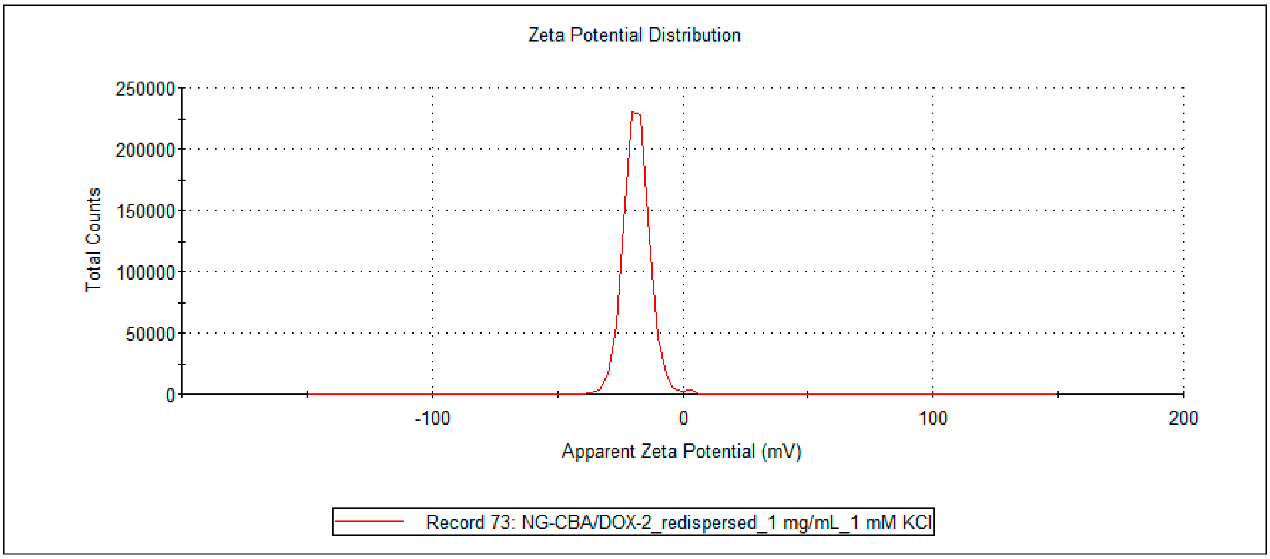

Figure S7. Average zeta potential of NG-CBA/DOX-2, (n = 4).

			Mean (mV)	Area (%)	St Dev (mV)
Zeta Potential (mV):	-11.3	Peak 1:	-10.7	100.0	2.99
Zeta Deviation (mV):	3.10	Peak 2:	0.00	0.0	0.00
Conductivity (mS/cm):	0.0729	Peak 3:	0.00	0.0	0.00

Result quality : Good

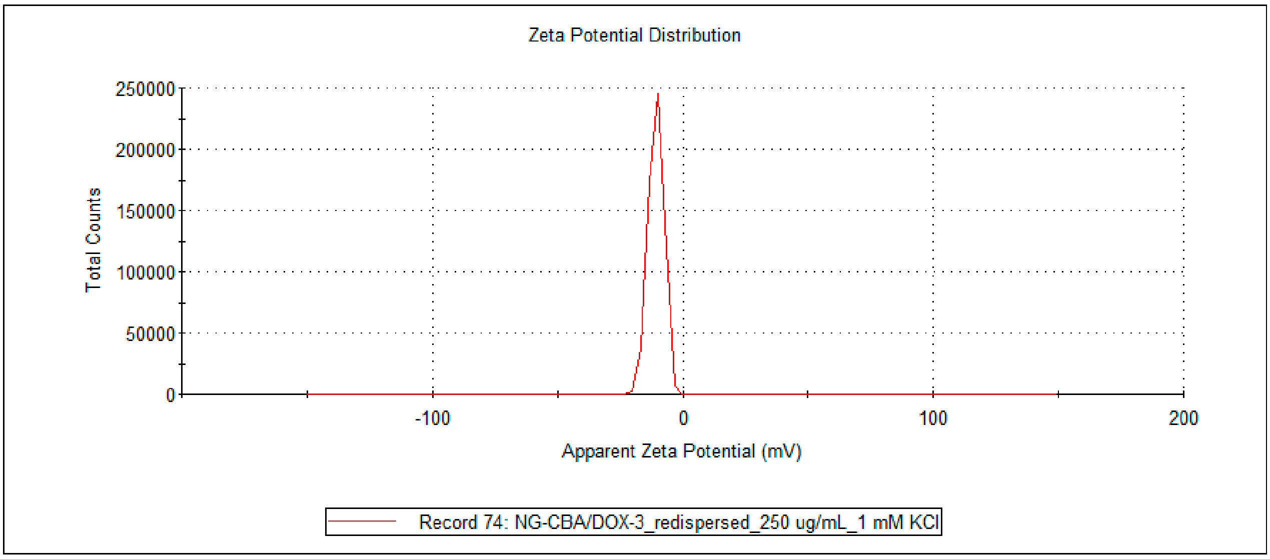

Figure S8. Average zeta potential of NG-CBA/DOX-3, (n = 4).
